# Perspective on the optics of medical imaging

**DOI:** 10.1117/1.JBO.28.12.121208

**Published:** 2023-09-30

**Authors:** Brian W. Pogue

**Affiliations:** University of Wisconsin–Madison, Department of Medical Physics, Madison, Wisconsin, United States

**Keywords:** optical, radiology, surgery, image, laparoscopy

## Abstract

**Significance:**

Medical imaging is very commonly described as synonymous with radiological imaging, yet optical imaging devices are widely distributed throughout many medical specialties. This delocalized nature of the technology reduces visibility and dominance as a cohesive medical technology sector.

**Aim:**

Indicators of impact of medical optical systems were examined and compared to the radiology technology sector.

**Approach:**

This study included a summary of (i) physician users, (ii) global technology valuations, and (iii) NIH funding levels. Analysis focused on comparing optical and radiological technology, comparing costs, funding, and finding differences, while tabulating strengths, weaknesses, opportunities, and threats to the field of optical imaging.

**Results:**

The 2023 global technology revenue valuation of biomedical optical tools is $128 billion USD/year while that of radiological tools is $48 billion USD/year. A direct comparison of US NIH funding in radiology shows $8.5 billion/year, whereas optical devices are nearer to $3.6 billion USD/year. R&D investment in applications, such as endoscopy, laparoscopy, and pulse oximetry, is far below those of radiological tools when normalized by valuation.

**Conclusions:**

The medical optical device industry is highly fragmented but has become the largest single technology sector in medicine today. When contrasted to radiology, it appears underfunded for research, where point-of-care tools such as surgery, endoscopy, laparoscopy, ophthalmology, pulse oximetry, and monitoring have more potential for development through research investment.

## 1 Introduction

Medical devices used in patient care have many forms, ranging all the way from multi-million-dollar imaging systems to guide interventional procedures, to pocket-sized diagnostic systems that cost a few hundred dollars. The major tools developed for imaging or intervention fall into two very broad categories, which can be roughly categorized as radiologic systems or optical systems. This is the central hypothesis examined in this paper that while radiological systems are well defined and organized around a small set of medical specialties and with a harmonized departmental representation. In contrast, optical systems are widely distributed and not clearly appreciated as a singular technology. This latter issue is even though optical devices permeate throughout nearly all medical specialties and compose an even larger and more highly diverse set of tools than radiological systems. Arguments could be made to classify technologies in different ways, but the breakdown of optical and radiological is examined here because these two have the majority presence in both diagnostic and therapeutic procedures and have very different representations, use cases, and market drivers. The analysis of how to compare optical systems to radiological systems is not obvious, but this study examines a few ways to think about comparing their numbers, value, and utility. An earlier version of this analysis was published in 2018,^1^ but this new version is updated with 2022 to 2023 numbers and an augmented analysis based upon current data.

Within medicine, “medical imaging” is nearly always defined as imaging with traditional radiological systems, such as x-rays, ultrasound, magnetic resonance imaging, and nuclear medicine methods. The uses of these imaging systems in radiology departments have shaped this narrative, defining what medical imaging is. This can be seen by the fact that nearly all textbooks on the subject and all departments of radiology, medical imaging, medical physics are exclusively organized around radiological systems, as defined above. This is further reinforced by the workflow in which most medical practitioners order radiological exams to be completed by the radiology department and interpreted by radiologists as a service. Radiology is now the largest revenue generator in most major medical centers, as the scan costs have stayed low while the need for them has seen dramatic increases. This organization of radiology as a unique specialty was, and remains, necessary due to the inherent risks of radiation and the skill set needed for interpretation of the images. There are deviations to this, such as in ultrasound, which are not inherently dangerous when used within its specified limits, and its use has spread to many other specialties, including family medicine. But in major medical centers, radiology is still a comparatively well-defined department with a common set of scanners that gets utilized as a service for many ambulatory, in-patient, and interventional exams. The companies that service these departments are focused on the departmental structure and purchasing goals. There is a common language, a collaboration pathway for academics, and specialists, physicians, academics, and industry professionals all attend the same conferences.

Comparing radiological device use and growth to the penetration and use of optical systems shows a very different adoption pattern, workflow, development landscape, and descriptive narrative about the field. Optical systems are highly diversified, and this diverse range of tools is distributed throughout medicine. Their use goes all the way from family practice through optometry, ophthalmology, dermatology, urology, surgery, etc., and often they are used in a continuous or functional manner, such as pulse oximetry. In fact, because optical devices are so diverse in their specialization and capabilities, there is little commonality between devices used in different medical specialties. This has led to a situation where it is challenging to find a global understanding of the magnitude of how much optical devices have impacted medicine. Again, while radiological devices tend to be highly centralized within a department, optical systems are scattered throughout medical centers and into family clinics, in a highly delocalized manner. The market forces for different optical devices are wildly different depending upon the department and the price point, which can span from a few hundred dollars in family medicine up to millions of dollars in surgical systems. It appears like there is little commonality of location in between physicians, academic developers, and industry professionals, where they attend different conferences and it seems like the disciplines do not have a high frequency of crosstalk between medical specialties. Thus it is hard to identify the entirety of the optical device world, because of its success in adoption throughout nearly all of medicine. It is not generally viewed as a single technology sector, but rather each specialty views optical devices as one of the many tools in its toolkit.

In this study, an analysis of the penetrance, use, and impact of optical devices was carried out, using radiological devices as a comparator. The goal was to quantify the field as much as is possible and to identify areas where visualizing it this way might lead to advances.

## 2 Medical Specialties and Optical Devices

**Fig. 1:**
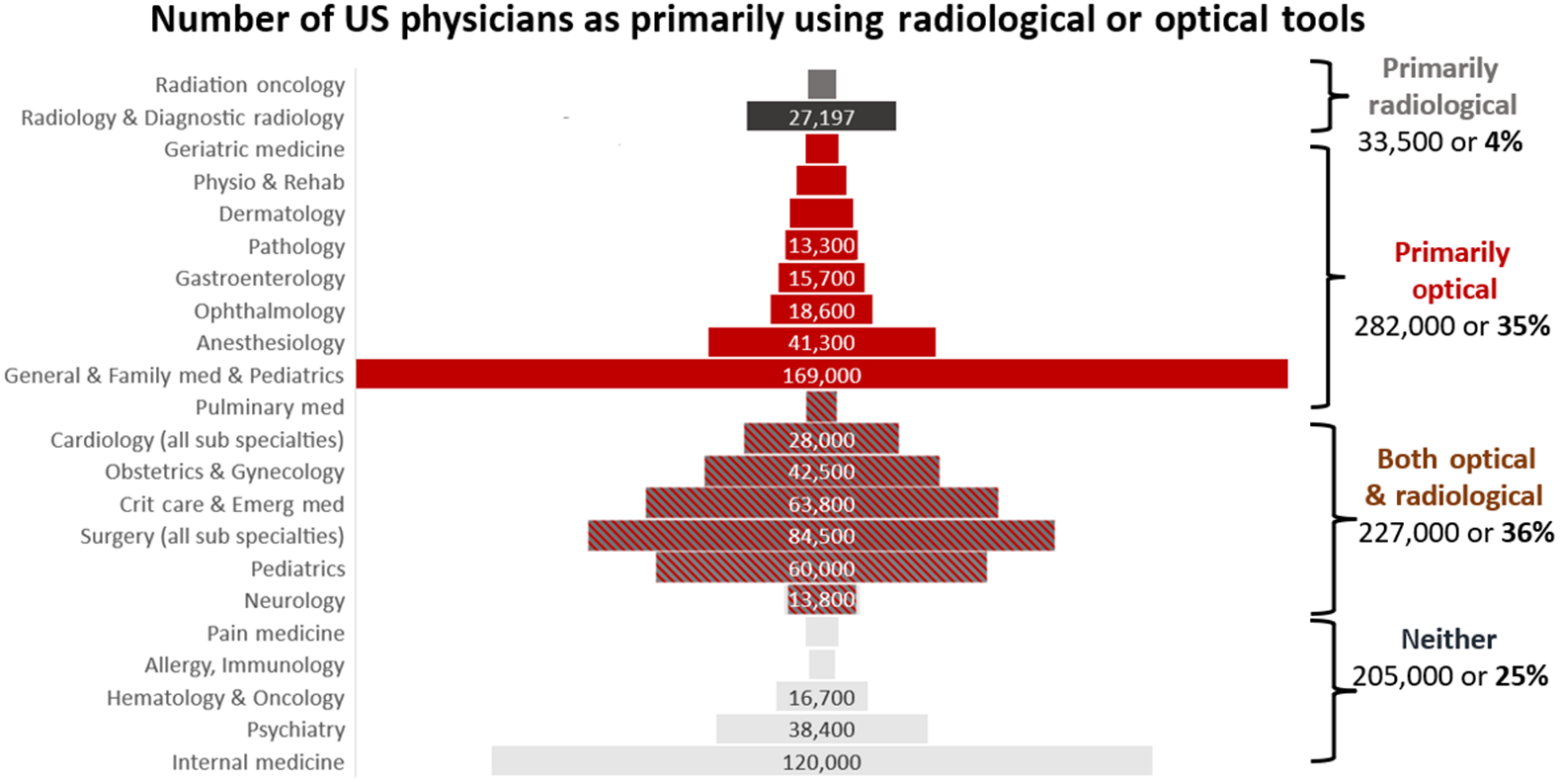
Numbers of physicians in the United States (from the American Medical Association in 2021)^2^ roughly categorized into those that primarily utilize radiological systems on a daily basis, those primarily using optical systems on a daily basis, those that utilize both, and those that utilize neither daily.

The United States has nearly 1 million physicians, according to the American Medical Association,^2^ and a breakdown of these into specialties based on their numbers is illustrated in Fig. 1. Those that utilize radiological tools (largely radiation oncology and radiology) make up about 4% of all physicians. The categorization of these two departments is the clearest, given their complete reliance on radiological systems. Those that utilize primarily optical tools are slightly less clear to classify, but are dominated by those general and family medicine who do not have radiological licenses in their office but can also including geriatrics, dermatology, gastroenterology, pathology, ophthalmology, anesthesiology, and physiology and rehab. Together these compose ∼35% of all physicians. There is a broad category of specialties that utilize both radiological and optical systems, and it is much more challenging to categorically say, but these including surgery, critical care, obstetrics and gynecology, cardiology, and pulmonary medicine, composing an additional 36%. The actual breakdown of utility is challenging to make out, so these are very broad categories. There are a range of physicians who have a high potential for not utilizing either of these devices, including internal medicine, psychiatry, hematology and oncology, allergy and immunology, and pain medicine, although they likely work with colleagues who do use both. However, when broken down by these broad approximate categories, likely 66% or a full 2/3 of all physicians utilize optical systems on a nearly daily basis, making it clearer that optical technology is the largest single technology utilized by physicians in the country.

**Fig. 2:**
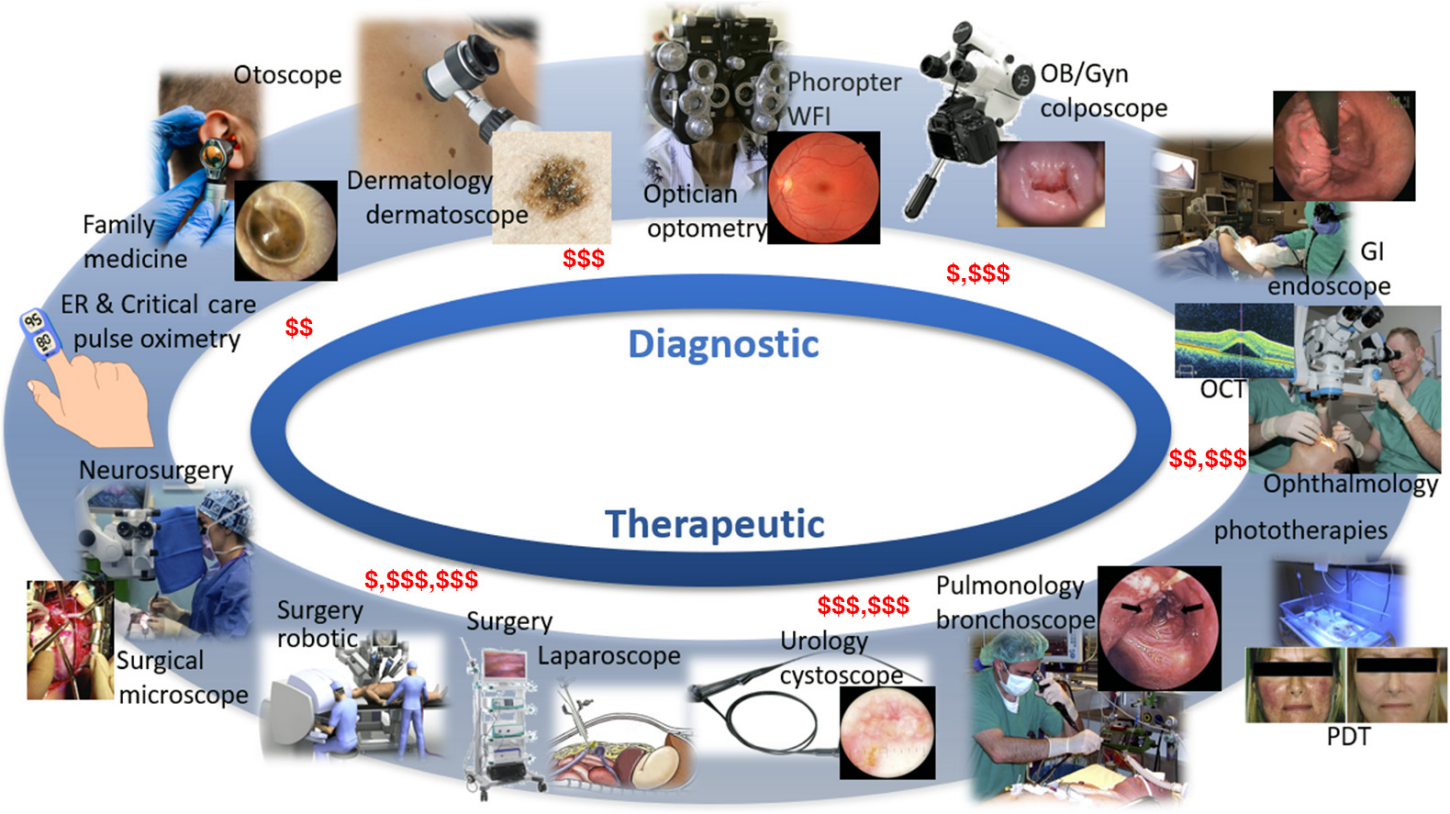
Visual display of a range of many commonly adopted optical technologies used in medicine, broadly classified as diagnostic (top) and therapeutic (bottom), and roughly organized clockwise from lower system cost (top left) to higher system cost (bottom left), as noted by red dollar signs.

Gaining a high-level perspective on optical systems in medicine is challenging, because there is a disconnect between academic biomedical optical system development and industrial medical system development. This disconnect is partly due to national funding trends, as will be discussed below, as well as the fact that large medical device companies substantially internalize their research programs. Thus the academic research in optical systems tends to be more biased toward scientific or fundamental developments, such as microscopy systems, whereas the device industry tends to focus on iterative improvements to highly profitable existing systems, which are largely macroscopic optics-based. A high-level overview of systems in widespread use can be found from researching the tools used by the physician specialties listed in Fig. 1. Figure 2 illustrates the range of tools that have been widely adopted and in turn shape what these specialties do. This is organized roughly around diagnostic tools on the top and cascading to therapeutic and therapy guidance tools on the bottom.

## 3 Global Valuation of Optical Devices

**Fig. 3:**
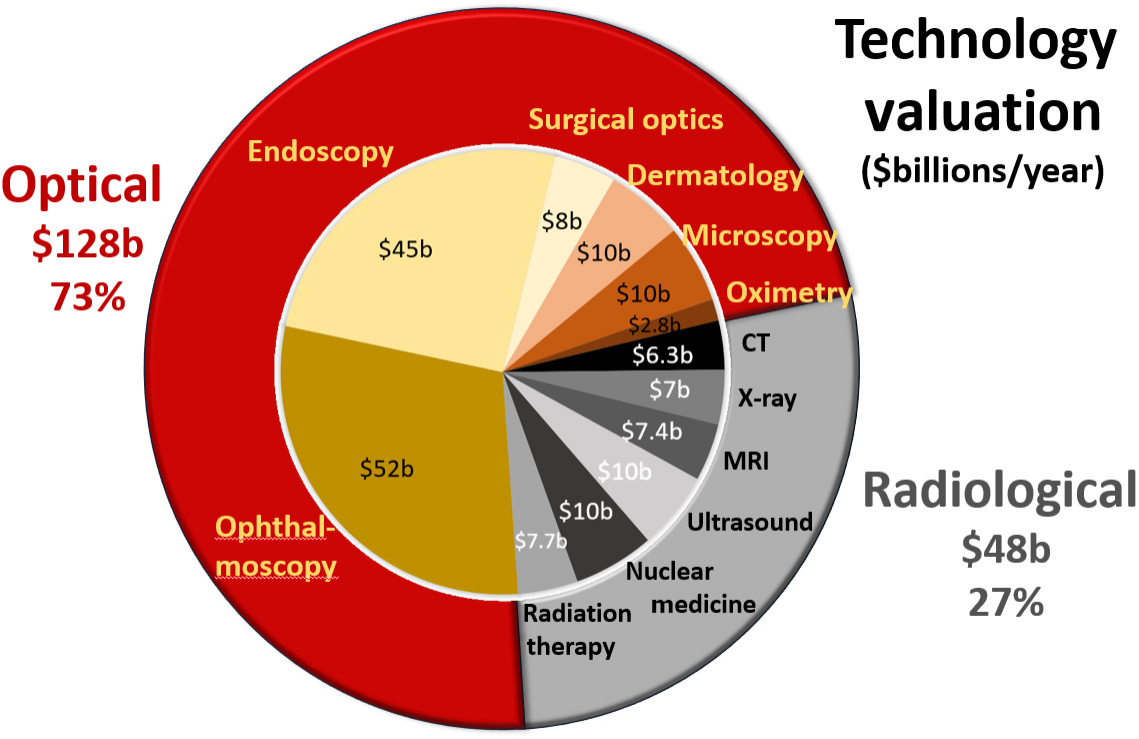
Global market valuation of six radiological areas (CT, x-ray, MRI, ultrasound, nuclear medicine, and radiotherapy) is summarized above totaling $48 billion for 27% of the global device market. The optical technology areas that were largest are also summarized (ophthalmology, endoscopy, surgery, dermatology, microscopy, and pulse oximetry) totaling $128 billion/year for 73% market share.

One of the most straightforward ways to achieve a top-level view of the impact of a technology is to assess its financial valuation, here defined as the market investment in each sector, as assessed by consulting reports. A survey of consulting reports was completed with at least three published reports assessed for each technology sector (see Table 1), and the average of these reports was summarized into a single global market value number for the 2022 to 2023 timeframe. The summary of these numbers is shown below in Fig. 3, illustrating the market impact of optical devices and radiological devices, organized into red and gray, respectively. It is interesting to note that the optical device valuation is 73% as compared to just 27% for radiological systems. The details of the reports utilized as resources and the total range of numbers are listed in Table 1.

**Table 1:** Complete listing of total range for global market valuation numbers for each modality from Fig. 3, with the listing of the market research report origin. Note some reports lack clarity about the inclusion of disposables or other non-device parts to their valuation, which can lead to high variation in the range, such as for dermatology.

Technology	Valuation range	Resources
Ophthalmology	$50b to $56b	GlobeNewswire
		Research and Markets
		PR Newswire
Endoscopy	$30b to $49b	Grand View Research
		MarketsandMarkets
		PR Newswire
Surgical devices	$5b to $17b	Research and Markets
		Grand View Research
		Expert Market Research
Dermatology	$6.5b to $23b	Grand View Research
		MarketsandMarkets
		Verified Market Research
Microscopy	$7.5b to $12b	Grand View Research
		MarketsandMarkets
		BCC Research
		Maximize Market Research
Pulse oximetry	$2.5b to $2.9b	Grand View Research
		MarketsandMarkets
		GlobeNewswire
		BioSpace
CT	$4.0b to $8.1b	Grand View Research
		Mordor Intelligence
		Fortune Business Insights
X-ray	$3.8b to $7.0b	Research and Markets
		Future Market Insights
		Mordor Intelligence
MRI	$5.3b to $9.0b	Grand View Research
		Statista
		Mordor Intelligence
Ultrasound	$8.9b to $10.8b	Grand View Research
		GlobeNewswire
		Mordor Intelligence
Nuclear medicine	$9.9b to $11.7b	Grand View Research
		Precedence Research
		Image Technology News
		Maximize Market Research
Radiotherapy	$5.7b to $8.6b	Research and Markets
		Precedence Research
		Grand View Research
		Global Market Insights

These global valuations are based upon industry numbers for total market manufacture and sales of systems annually. Notably this does not represent the revenue seen at medical centers for use of the systems, which is different. For example, it is well known that radiology departments are one of the highest profit-generating medical centers in the United States, and this may be partly because these are diagnostic procedures that are rapid, with many scans per day per system. The billing costs for imaging and interpretation of the images is in the range of $20 billion per year, from about 370 million procedures, with a low reimbursement per exam near $100. This can be contrasted with an optically guided procedure, such as laparoscopy, of which there are about 15 million/year in the United States, and revenue is near $10,000 per procedure, for a comparatively modest revenue of $150 million/year for laparoscopy. So, while diagnostic imaging has low reimbursements, they can have higher financial impact on the medical center because of the larger number of procedures done and the number of scans per day that can be achieved on a single system. Diagnostic procedures also wind up with diagnoses of health conditions that often will lead to further treatments, further increasing revenue for that medical center. Interventional procedures require substantial cleaning and sterilization of devices, which increases the cost per procedure and may require more systems to be present in a single institution.

## 4 US Investment in Research on Optical Devices

**Fig. 4:**
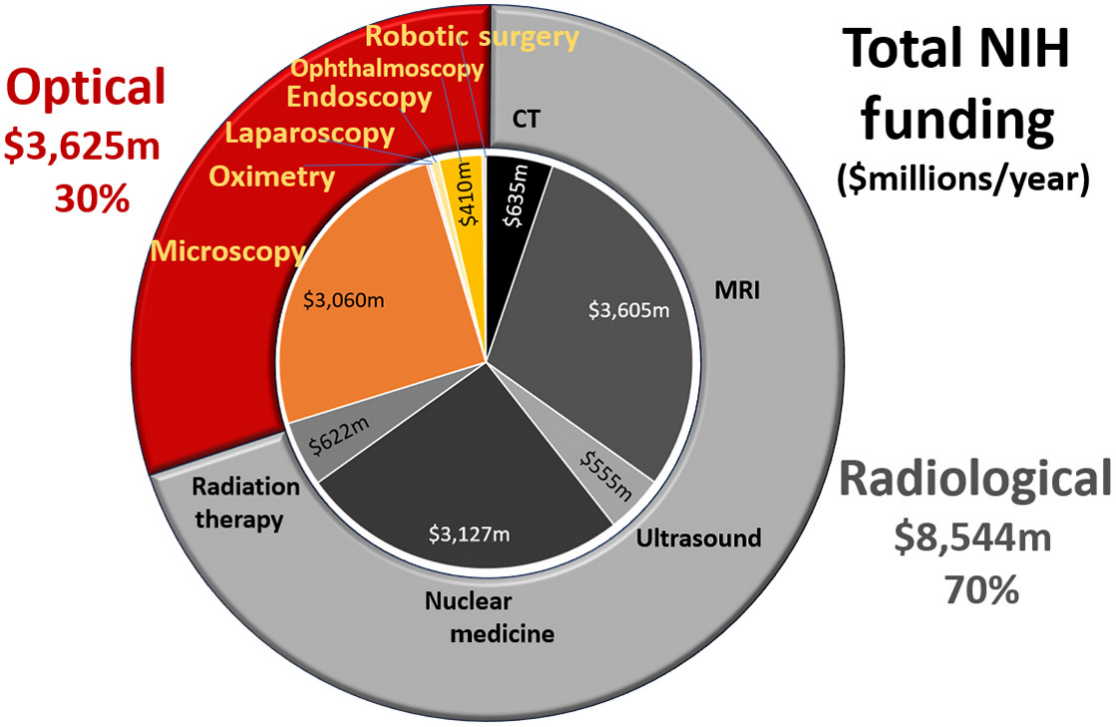
NIH funding levels for 2023 were surveyed from NIH RePORTER,^3^ with total dollars of funding listed, in millions of USD. The levels for some are too small to show in the chart. Optical tools make up $3.6 billion while radiological tools make up $8.5 billion in funding per year.

Another important factor that is involved with device development is the amount of investment in research and development. It is challenging to assess this from company information, but public disclosure of funding from the United States National Institutes of Health (NIH) is available. The NIH RePORTER system^3^ was queried to count the number of grants and dollars devoted to funding in key radiology and optical technology areas, and this is displayed in Fig. 4. In the radiological sectors, this was broken into x-ray CT, MRI, ultrasound, nuclear medicine, and radiotherapy, for a total of $8.5 billion per year, or 70% of the device-based research found. In the optical sectors, this was broken down into microscopy, endoscopy, pulse oximetry, laparoscopy, ophthalmology, and robotic surgery, for a total of $3.6 billion per year, or 30% of the device-based research found. It is very interesting to reflect upon the mismatch in balance between the revenue-based valuation of technology from Fig. 3, and this investment in research found in Fig. 4.

If the technologies are analyzed as a factor of R&D investment per revenue valuation, there are some surprising observations, as shown in Table 2. Several key technologies that are widely used in scientific study have high funding rates, including MRI, nuclear medicine, and microscopy, with above 30% funding per valuation of the technology. This is an amazingly high funding rate and indicative of the scientific value of their advancement as much as their clinical utility. Older imaging modalities and those that do not have large scientific discovery value tend to have lower NIH funding rates, including CT, ultrasound, and radiotherapy. These might also include static technologies, such as pulse oximetry, despite how widespread and clinically meaningful the technology is for critical care. However, there are some optical technologies that have surprisingly low investment in R&D given their valuation and these include ophthalmology, and especially laparoscopy and endoscopy. These latter two areas are highly interventional but also are used in a point-of-care setting where their use has direct curative intent and cannot be replaced. It could be argued that further investment in these technologies is warranted. Why research into these devices does not have larger funding at government agencies like the NIH is not clear, but this should be a topic of further analysis.

**Table 2:** Ratio of NIH funding to total global valuation of the technology is shown for the range of radiologic and optical tools where data matched.

Imaging device	Funding ratio (%)
CT	10.1
MRI	48.7
Ultrasound	5.6
Nuclear medicine	30.1
Radiotherapy	8.1
Microscopy	30.6
Pulse oximetry	0.8
Laparoscopy	0.2
Endoscopy	0.2
Ophthalmology	0.8

**Table 3:** Strengths, weaknesses, opportunities & threats (SWOT) of the technology sector of medical optical imaging.

Strengths	Weaknesses
• Highly efficient economical imaging due to invention and advancement of CMOS cameras	• Largely limited to surface and cavity imaging, vision imaging, or near sub-surface sensing/imaging
• Enormous economy of scale with consumer optical devices, a $2.8 trillion industry	• Tissue imaging deeper than a few mm has never been commercially successful due to limited spatial resolution
• Large engineering workforce, largest technology sector in medicine	• Each application has specialized system, diffusing the overall view of optical imaging systems
• Primary tool for point-of-care and interventional vision	• Slow introduction of contrast agents
• Optical devices are core to many highly sensitive radiation detection systems	
• Synergy with advances in display technologies	
Opportunities	Threats
• Continuing explosion of consumer technologies that advance optical imaging capabilities	• Dearth of communication between academic and industrial biomedical optics research directions
• Advanced surgical, laparoscopic, endoscopic technologies that augment vision beyond just color imaging	• Limited NIH investment in optical tech despite being widely adopted in point-of-care exams
• Microscopic to macroscopic imaging in the same instruments	• Funding structures limit the ability to work with industry on collaborative development
• Scattering makes the signal more sensitive to the entire tissue volume, beyond vessels	• Lack of technical domain experts within the medical center to assist physician groups when deploying advanced medical optical systems
• Highest potential for molecular sensing of all imaging modalities due to numerous optical molecular probes	• Physician work overload minimizing use of advanced instruments
• Shifting healthcare toward wellness instead of healthcare, requiring monitoring technologies	
• Screening requires low-cost, low-risk systems	

## 5 Analysis of Strengths, Weaknesses, Opportunities, and Threats

To augment the data below, a narrative summary of strengths, weaknesses, opportunities, and threats (SWOT) provides a structured way to summarize the status of the field and help point toward areas that are ripe for development. The details of these are necessarily slightly subjective, but the attempt has been made to be objective and provide an overview perspective, without getting into details of any modality or procedure. A summary of the SWOT details are included in Table 3, and a narrative description of each point is included below in this section.

### 5.1 Strengths

The largest technological strength of optical imaging can be clearly referenced to the invention and enormous advances in CMOS camera sensors in the last 25 years.^4^ These devices now widely outnumber the number of humans on the planet and are ubiquitous in everyday life, from cell phones and computers, to every digital image that we see daily. Because of economy of scale, very high-end sensors can be just a few dollars in cost and even disposable in their use case. This in turn has led to an enormous optical device industry throughout all sectors of the economy, a $2.5 trillion industry composing 3% of the >$85trillion global economy. Biomedical optics is actually a fairly small sector, here estimated at $128 billion, increased from $91 billion in 2015.^5^ So biomedical optics is 0.15% of the entire global optics industry, but this means that it leverages the economy of scale that is 700 times its size. This scale reduces the cost of components enormously, making components available at a lower cost than if they were just produced for the smaller market of medicine. Within medicine though, optics forms the largest technology market in medical imaging, and many of the tools and detectors for optical sensing are still the core technology used in high-end nuclear medicine scanners, advancing technologies, such as CMOS arrays and silicon photomultiplier tubes.

Perhaps the most obvious medical strength of optical systems is through their use as the primary tool for all point-of-care vision, where the physician and the patient are in the same room.^6^ The physician’s desire is to diagnose by seeing inside the body, through ear, eye, nose, throat, rectum, colon, vagina, urethra, or skin during a clinical exam, partly illustrated in Fig. 1. Optical cameras augment their vision with better tools that fit into the body cavity and deliver high-fidelity images. However substantial improvement may still be possible, such as the ability to provide more wavelength bands, higher resolution to the point of microscopy, molecular sensing, and tissue response imaging. Innovations to be developed and tested in clinical trials will determine the success of these.

### 5.2 Weaknesses

The weaknesses of optical imaging limit what is possible today and in the future. The most obvious limitation is that optical light is highly scattered in tissue, which relegates it to primarily surface imaging throughout the skin, ears, eyes, or mucosal cavities of the body. Although not necessarily a weakness, this trait defines where optical imaging provides its maximum value. It is superior to any other tool for surface imaging. Conversely, it is true that deep tissue imaging of more than a millimeter or two has never been commercially successful due to limited spatial resolution. Sensing through tissue such as in pulse oximetry is very successful but imaging has not been. Although significant advances have occurred in deep tissue sensing and imaging with approaches like diffuse optical tomography and photoacoustic imaging, these have not translated beyond research systems into clinical practice. In comparison to imaging though, there are many good applications for sensing through tissue, such as pulse oximetry. But true imaging with spatial resolution has not been commercially adopted in medicine today, and given the timeline of development and substantial testing, it seems likely to follow in the future as well.

A weakness inherent in optical systems being interventional or direct point-of-care is that each new application has a specialized system and as shown in Fig. 1, there can be very little commonality between systems in different specialties. As such, this extreme diversification diffuses the broader view of what optical imaging technology is within medicine or medical imaging. Working in ways to better define these systems is something that professional societies might work on to better represent the field of biomedical optics.

### 5.3 Opportunities

Key technological opportunities for optical imaging lie in simply harvesting from the continuing explosion of consumer technologies that advance optical imaging, which keeps producing more features, capabilities, with embedded processing and expanded spatial resolution, wavelength range, dynamic range, low noise, and expanded size scales. The advances in sensors and packaged systems do not necessarily directly translate to medical use, but the wider advancement of the field creates a technology sector that can invent tools with lower cost production. This will allow major advances in areas, such as surgical, laparoscopic, and endoscopic technologies that augment vision beyond just color imaging. Additionally, incredible opportunities lie in combining microscopic to macroscopic imaging in the same instruments, allowing point of care or interventionalists to utilize both these size scales. Optics has always been good at both magnification scales but rarely has been good at blending the two.

Opportunities remain undeveloped in the areas of molecular sensing in medicine. This aspect of optics is widely utilized *in vitro* through millions of microscopy techniques, molecular pathology, flow cytometry, gene profiling, and clinical chemistry. However, many of these tools are not used *in vivo* because of the limits to their medicinal chemistry use, despite being incredibly important for healthcare. Advancement of surgical trials or diagnostic trials with well-tolerated molecular contrast agents is an area that is ripe for advancement. An additional feature of optical sensing through tissue is that while multiple scattering reduces the ability to image with high resolution, it does increase the sensitivity of tissue, by increasing the pathlength in tissue by 5 to 6 times,^7^ enhancing the sensitivity to cells and capillaries. So non-image-based sensing of molecular and capillary features can be significantly improved with highly scattered light. This is a factor exploited in pulse oximetry sensing^8^ but not widely exploited yet for other applications that have more molecular features.

Future healthcare opportunities lie in the ongoing shifts in the funding and goals of healthcare toward wellness and health monitoring, instead of acute delivery of healthcare once problems emerge. The commercial monitoring tools developed for home sensing and daily monitoring are coming from the consumer electronics industry, in which optical sensing is a key core technology. The costs of these technologies, and those for screening for healthcare problems, each require a low-cost, low-risk system, and optical technologies are likely going to be a core part of this pipeline.

### 5.4 Threats

One of the largest threats to the advancement of optical technologies in medicine comes from the fact that academic research and industry research are not at all aligned in goals or even in any realistic communication with each other. Academic research is funded by government agencies such as NIH, which advance scientifically intriguing technology, but the optical device industry tends to be insular, inventing their own technologies within each company to advance new products. This is partly because the field is so mature, and so there are thousands of overlapping patents in each field, leading to the development space being just as much about trade secrets and proprietary software as it is about innovations in hardware technology. Thus communication and advancement are hindered in this environment, and the dynamics do not lead to collaborative development. Unlike the radiological field, where industry, academics, and physicians often attend the same conferences together (i.e. Radiological Society of North America, and the American Society for Therapeutic Radiation Oncology) and work on the same devices, the optical device sector and people are highly diversified into their physician specialty areas. This means that there is little communication across device specialties, such as dermatology, surgery, cardiology, family medicine, and oncology. Solving this communication and impedance mismatch problem would have positive potential for technological advancement across medicine and provide better guidance. It also may help better align funding deficiencies in certain areas of research. The need for this is illustrated by the low funding for surgery, laparoscopy, and endoscopy, as shown in Table 2, despite their critically important role in medicine today.

A medical threat can be seen also in the comparison between biomedical optics and radiology. Most radiology and radiation oncology departments have entire subspecialties of medical physicists within their departments, providing them with technical expertise, repair, installation, and commissioning of new advanced devices. In comparison, optical devices are rarely ever deployed with technical guidance beyond the company sales or installation specialist, and training is either remote or not at all in some cases. Thus as optical technologies become more advanced, there is increasing potential for misuse or early adopters to fail in the accurate or informed use of the technology. There is a challenge that advanced highly technical areas may need to advance a field of optical engineers or physicists as a resource for people across the medical specialties. This occurs in some of the most research active medical centers now but is not common throughout most medical centers. Finally, there is some risk that high-end technology will limit its advance in routine medicine because of the growing overload of work, documentation, and billing inherent in the system. Physician burnout will inevitably reduce use of technology, limiting future innovation adoption.

## 6 Discussion

Part of the rationale for this analysis was the fact that optical technologies are so diverse and spread across so many medical specialties, such that it is hard to grasp the size and scale of the technology sector. The contrast of optical with radiological was developed to illustrate how even a distributed set of unrelated technologies (i.e., CT, MRI, ultrasound, and nuclear medicine) can be homogeneously represented in a single department, radiology. This physical co-localization and user base homogeneity works to its advantage to highlight the field as a whole and simplify the ability to integrate company collaborations at a high level, as well as national funding streams and integration of academic developers. Optical systems are highly divergent, stratified along specialization lines and along price point goals, with comparatively little crosstalk in the user base. Companies can translate system innovations across specialization areas, but this is not often done in constructive collaboration with the users nor academic developers. Additionally, because of the distributed nature of optical systems, the industry base is also highly distributed, and the academic base of researchers does not necessarily match that of industry. Industry has developed its own R&D program for most interventional systems, and there is a direct market from companies to specialists without involvement of academics. The lack of visibility is part of the reason optical devices are not seen as a single technology sector today, and it is compounded by the distributed nature of medical specialties within separate departments or divisions.

Communication barriers are also a factor that limits the development and realization of optical imaging as a cohesive technology sector. These barriers are because of this distributed specialty nature, and the fact that academic developers rarely meet with the users. This can be contrasted with radiology, where the academic developers and users are commonly located in the same academic medical center or department allowing direct communication and collaboration on research projects. Also the radiologists are trained on the whole realm of radiological tools, even if they eventually specialize in one of them, they are trained on the whole sphere of imaging and therapy approaches in radiology. This can be contrasted with the optical users in surgery, ophthalmology, dermatology, and pathology, where there is little communication on common tools. Additionally, fewer optical academic researchers are embedded into these departments at most centers, as compared to large radiology departments that have robust academic research programs. Thus optical device development suffers from multiple communication barriers that limit lateral translations and vertically driven innovations in technology. This aspect is compounded with the other factors to limit the potential of the field.

The most expensive optical technologies are interventional and exist within medical specialties that tend to have less research because of their interventional or procedure-based approach to practice. These include gastroenterology, surgery, and dermatology, for example, where the funding rate and number of controlled clinical trials is less than medicine or medical oncology. Still, diagnostic radiology has been able to develop research centers around the country that advance the tools of imaging, partly because of their promise, but also partly because of their visibility and cohesive approach to advancing the field as a unified technology sector. A concrete example of this cohesion is shown in the fact that NIH National Institute of Biomedical Imaging and Bioengineering funded P41 centers for Biomedical Imaging, where 10 are focused on MRI or PET, and only 3 are focused on optical technologies in medicine. This is partly because the diversity of optical technologies makes it harder to exploit a common platform but also partly because radiological systems represent singular large investments in technology that work as research resources that attract users. It is worth speculating that if surgical departments put their same focus on the commonality of high-fidelity higher-parametric functionality imaging systems and advancing them as a technology sector that higher investment and advancement could follow.

## 7 Conclusions

This analysis had the goal of providing quantitative numbers on the market forces of optical technology and the user base of physicians. It illustrates that optical devices do form the single largest technology sector and larger than that of radiology, in terms of purchased equipment. It is important to note that radiology still has a very high profit margin, making it the largest profit center in most medical centers, but this comes from the higher volumes of exams per day on a limited set of imaging systems. The optical technologies used in many point-of-care procedures have significant value and when viewed as a technology sector may be better positioned to translate ideas or find innovations that advance the tool capabilities. Research in the space of larger interventional guidance systems, such as laparoscopy and endoscopy, is significantly underfunded, given their position in medicine and the current NIH record on grants. Further insight into the scope of biomedical optical systems and ways to synergize across disciplines and enhance areas of needed development may benefit from this basic study.

## References

[1] Pogue B. W., “Optics of medical imaging,” SPIE Professional, 22 January 2018, https://www.spie.org/news/spie-professional-magazine-archive/2018-january/optics-of-medical-imaging

[2] “Physician specialty data report,” 2021, https://www.aamc.org/data-reports/workforce/data/number-people-active-physician-specialty-2021

[3] “NIH RePORTER,” 2023, https://reporter.nih.gov/advanced-search

[4] “CMOS sensors enable phone cameras, HD video,” in Consumer Goods, Spinoff N., Ed., (2017).

[5] Anderson S. G., “SPIE industry analysis: SPIE analyzes the biophotonics-enabled industry,” SPIE Professional, 1 April 2015, https://spie.org/news/spie-professional-magazine-archive/2015-april/industry-analysis-

[6] Boppart S. A. Richards-Kortum R., “Point-of-care and point-of-procedure optical imaging technologies for primary care and global health,” Sci. Transl. Med. 6(253), 253rv2 (2014). STMCBQ 1946-6234 10.1126/scitranslmed.3009725 PMC4370289 25210062

[7] Duncan A. et al., “Optical pathlength measurements on adult head, calf and forearm and the head of the newborn infant using phase resolved optical spectroscopy,” Phys. Med. Biol. 40(2), 295–304 (1995). PHMBA7 0031-9155 10.1088/0031-9155/40/2/007 7708855

[8] Wukitsch M. W. et al., “Pulse oximetry: analysis of theory, technology, and practice,” J. Clin. Monit. 4(4), 290–301 (1988). JCMOEH 0748-1977 10.1007/BF01617328 3057122

